# Proteomic profiling of archaeological human bone

**DOI:** 10.1098/rsos.161004

**Published:** 2017-06-07

**Authors:** Rikai Sawafuji, Enrico Cappellini, Tomohito Nagaoka, Anna K. Fotakis, Rosa Rakownikow Jersie-Christensen, Jesper V. Olsen, Kazuaki Hirata, Shintaroh Ueda

**Affiliations:** 1 Department of Biological Sciences, Graduate School of Science, The University of Tokyo, 7-3-1 Hongo, Bunkyo-ku, Tokyo 113-0033, Japan; 2 Department of Human Biology and Anatomy, Graduate School of Medicine, University of the Ryukyus, 207 Uehara, Nishihara, Nakagami, Okinawa 903-0215, Japan; 3 Centre for GeoGenetics, Natural History Museum of Denmark, University of Copenhagen, ster Voldgade 5–7, 1350 Copenhagen, Denmark; 4 Department of Anatomy, St. Marianna University School of Medicine, 2-16-1 Sugao, Miyamae-ku, Kawasaki, Kanagawa 216-8511, Japan; 5 Novo Nordisk Foundation Center for Protein Research, Faculty of Health Sciences, University of Copenhagen, Blegdamsvej 3b, 2200 Copenhagen, Denmark; 6 School of Medicine, Hangzhou Normal University, No.58, Haishu Road, Cangqian, Yuhang District, Hangzhou, Zhejiang 311121, People’s Republic of China

**Keywords:** proteomics, immune system, age-related changes, archaeological bone, mass spectrometry

## Abstract

Ancient protein analysis provides clues to human life and diseases from ancient times. Here, we performed shotgun proteomics of human archeological bones for the first time, using rib bones from the Hitotsubashi site (AD 1657–1683) in Tokyo, called Edo in ancient times. The output data obtained were analysed using Gene Ontology and label-free quantification. We detected leucocyte-derived proteins, possibly originating from the bone marrow of the rib. Particularly prevalent and relatively high expression of eosinophil peroxidase suggests the influence of infectious diseases. This scenario is plausible, considering the overcrowding and unhygienic living conditions of the Edo city described in the historical literature. We also observed age-dependent differences in proteome profiles, particularly for proteins involved in developmental processes. Among them, alpha-2-HS-glycoprotein demonstrated a strong negative correlation with age. These results suggest that analysis of ancient proteins could provide a useful indicator of stress, disease, starvation, obesity and other kinds of physiological and pathological information.

## 1. Introduction

Ancient proteins have been studied with the aim of revealing the life and diseases of past humans, species identification and evolution [1–3]. Immunological methods were applied to archaeological human bones as early as the 1980s [4]. Haemoglobin, serum albumin (ALB) and immunoglobulin G (IgG) were studied using immunological methods for species identification and for detecting immune responses [5–7].

In recent years, proteomics analysis by liquid chromatography tandem mass spectrometry (LC-MS/MS) has attracted more attention owing to its accuracy and robustness and to the remarkable improvement in instruments and protein databases; this method has also been applied to ancient remains [8–11]. The first LC-MS applied to archaeological remains was conducted by Schweitzer *et al.* [12]. Then shotgun proteomics analysis was performed on a 43 000-year-old woolly mammoth bone [8]. Since then, the method has been applied to other species [9–11,13–16]. With regard to archaeological human bones, ancient protein analyses have been performed to detect disease and immune response [17,18]; however, to date, shotgun proteomics analysis has not been used to profile the bone proteome of archaeological human remains. In this study, we present the results of shotgun proteomic profiling of archaeological human bones. We performed shotgun proteomics of human remains using eight rib bones excavated from the Hitotsubashi site of the Edo period, expecting to acquire physiological information.

We used rib samples from eight skeletons excavated from the Hitotsubashi site (AD 1657–1683) in Tokyo, Japan. The Hitotsubashi site is located in the city of Edo, the former name for Tokyo, and is known for its rapid urbanization and stressful living environment [19–21]. Our findings suggest that the bone proteome represents a source of information about immune system activity and age-related changes. Our work demonstrates that the proteomes of archaeological bone represent a source of biomolecular information for the reconstruction of physiological and pathological processes that occurred in antiquity but cannot be detected using other bioarchaeological methods.

## 2. Material and methods

### 2.1. Human bone samples

Two infants and six adults, including four elderly adults (more than 60 years old), were obtained from the skeletal collection of the Hitotsubashi site (AD 1657–1683) in Tokyo, Japan. The chronological age was determined on the grounds of the stratigraphic layer, which is between the Great fire of Meireki (AD 1657) and the fire of Tenna (AD 1684). The individuals buried in this graveyard are townspeople, known from the fact that the largest proportion of graves consisted of the wooden coffins typically used for the lower social classes. Nagaoka & Hirata [21] had reported the sex and age at death of the adult skeletons used in this study. Sex was determined by the sexual dimorphism of the pelvis using the dimorphic criteria of the pelvis including the greater sciatic notch [22–24], preauricular sulcus [23–25], ventral arc [26], subpubic concavity [26] and medial aspect of the ischiopubic ramus [26]. Age at death for each individual was estimated by the observation of the auricular surface of the ilium using the method of Buckberry & Chamberlain [27]. The auricular surface of the ilium is resistent to decay in forensic and archaeological contexts, and it seems to cover a wide range of ages up to elderly individuals [28]. The age estimation at death of adults aged more than 15 years was based on the chronological metamorphosis of the auricular surface of the ilium. Buckberry & Chamberlain [27] developed a quantitative system of 5–19 composite scores according to five morphological traits of the auricular surface: transverse organization, surface texture, microporosity, macroporosity and apical change. The 5–19 composite scores of the five traits are then classified into seven stages. Each obtained stage of the auricular surface of the ilium is then transformed into an average age at death with standard deviation shown in the data of Buckberry & Chamberlain [27]. Although the age at death estimation is more difficult in adults than in sub-adults, Murlhern & Jones [29] tested the reliability of the revised method using 309 individuals from the Terry Collection and detected that the Buckberry and Chamberlain’s method [27] improved the accuracy of age estimation for elderly adults. The age at death of sub-adults was estimated by the degree of formation and eruption of teeth [30,31].

### 2.2. Protein sample preparation

In order to prevent contamination, surfaces were removed from bone samples with sandpaper. Samples (30–50 mg) were wrapped in clean aluminium foil and fragmented into powder using a conventional hammer. The hammer was cleaned with bleach and ethanol, repeatedly. Powdered bone samples were washed three times with 300 μ*l* 0.5 M EDTA (pH 8.0), followed by incubation at 4 ^°^C for 24–48 h with agitation. Samples were washed again three times with 100 μl 0.1 M Tris (pH 8.0). Samples were suspended in 300 μl 6 M guanidinium hydrochloride (GuHCl), 10 mM Tris (2-carboxyethyl) phosphine, 20 mM chloroacetamide and 200 mM Tris (pH 8.0). Soft organic pellets eventually released after demineralization were disrupted using single-use individually sealed disposable micropestles (Eppendorf), cleaned before use with a 5% bleach solution and 95% ethanol (v/v). Without separating supernatants and the residual bone powder, samples were heated at 80 ^°^C for 2 h, and cooled to room temperature. LysC-Trypsin mix, 1/100 by amount of protein, was added to the samples for protein digestion. Mixed samples were incubated at 25 ^°^C for 30 min then diluted to 2 M GuHCl with 25 mM Tris (pH 8.0), followed by incubation at 37 ^°^C overnight with agitation. Digestion was terminated with 10% trifluoroacetic acid to a final concentration of 1%. After centrifugation at 14 000 *g* for 10 min, the tryptic peptides in the supernatant were immobilized on C18 stage tips as previously described [8]. Peptide mixtures were analysed by online nanoflow reversed-phase C18 LC-MS/MS, as described previously [32]. Briefly, peptides were separated on a 50 cm PicoFrit column (75 mm inner diameter) in-house packed with 1.9 mm C18 beads (Reprosil-AQ Pur, Dr. Maisch) on an EASY-nLC 1000 system connected to a Q-Exactive HF (Thermo Scientific) on a 165 min gradient. The Q-Exactive HF was operated in data-dependent top 10 mode. Full scan mass spectra were recorded at a resolution of 120 000 at *m*/*z* 200 over the *m*/*z* range 300–1750 with a target value of 3×10^6^ and a maximum injection time of 20 ms. HCD-generated product ions were recorded with a maximum ion injection time set to 108 ms through a target value set to 2×10^5^ and recorded at a resolution of 60 000 with a fixed first mass set to *m*/*z* 100.

### 2.3. Data analysis

MS/MS spectra in ‘.raw’ file format were processed with Maxquant v. 1.5.3.28 [33] and Andromeda [34] against the human reference proteome (UniProtKB, downloaded on 13 December 2015). In every search, spectra matched against the common contaminants database were removed except ALB, a common protein in bones [35].

Although bovine serum albumin (BSA) can be a common contaminant in proteomics laboratories, it has been previously demonstrated that endogenous ALB from extinct species can be confidently retrieved from ancient bones [8,16,35]. We validated the authenticity of the human serum albumin retrieved in this study by checking for the absence of BSA peptides in all the samples. All but one peptide of ALB had the same sequence as human, not bovine (the one peptide sequence was the same as both human and bovine). This indicates that the contamination of BSA had little impact, if any. We also checked the evidence of deamidation in ALB peptide, so we included ALB in data analysis.

Cysteine carbamidomethylation was used as a fixed modification. Oxidation (M and P), Gln → pyro-Glu (N-term Q), Glu → pyro-Glu (N-term E) and deamidation (N and Q) were used as variable modifications. The peptide tolerance, in the main search, was set to 5 ppm, while the tolerance for MS/MS match was set to 20 ppm. Up to seven modifications per peptide and up to two missed cleavages were allowed. The false-discovery rate was set at 1%. Other parameters were used as those pre-set in the software. The first protein of major protein IDs was used as a protein name. The deamidation rate of collagen (COL1A1 and COL1A2) was calculated as the total number of deamidated glutamine residues divided by the total number of glutamine residues. The mass difference between the deamidated monoisotopic peak and the 13*C* peak of the amidated form of the same peptide is 19.34 mDa. Previous studies demonstrated that the use of an orbitrap mass spectrometer with high mass measurement accuracy and high resolving power, like the one used in this study (Q-Exactive HF), and a peptide tolerance value like the one we imposed, 5 ppm, enable correct assignment of deamidated peptides [36].

In order to compare the amount of proteins among samples, detected proteins were quantified using the exponentially modified protein abundance index (emPAI) score [37]. The emPAI score is calculated using the number of detected peptides normalized by the number of theoretically observable ones. The emPAI score of basic salivary proline-rich protein 3 (PRB3) was quite high because the number of theoretically observable peptides is two, which is too small for calculating emPAI. We therefore excluded this protein. The emPAI scores of the remaining proteins were normalized, respectively, by the score of collagen (COL1A1). In order to detect proteins that correlated with age, Pearson’s correlation analysis between age and normalized emPAI score was performed on the proteins that included more than 10 unique peptides in total samples. As multiple comparisons were performed, Bonferroni’s correction was used for correlation analysis. We used the corr.test and p.adjust functions in R for statistical analysis.

To reveal protein function, we used the Gene Ontology (GO) term. The PANTHER statistical over-representation test [38] was conducted to identify functional GO annotations that were enriched in each individual. We used the ‘GO experimental only’ database for reliable annotation. Focusing on the fold change of proteins associated with developmental function, we calculated the correlation test between age and fold change of proteins that have the ‘developmental process’ term (GO: 0032502). We also used the STRING database [39] to create a network of functional relationships between the identified top 30 proteins.

## 3. Results

### 3.1. Proteins detected from archaeological human bones

**Figure 1.**
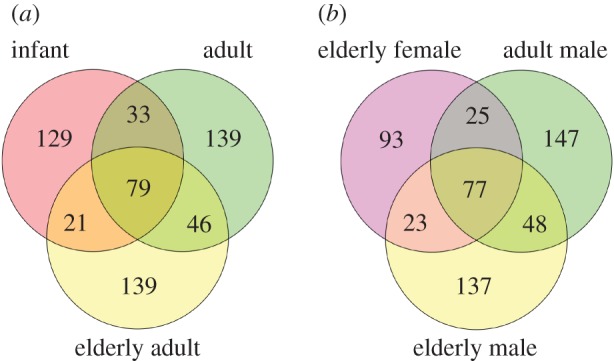
Venn diagrams of proteins between (*a*) life stages and (*b*) sex. (*a*) Infant: H-9 and H-142; adult: H-91 and H-206; elderly adult: H-88 and H-160. (*b*) Adult male: H-91 and H-206; elderly female: H-24 and H-162; elderly male: H-88 and H-160.

**Figure 2.**
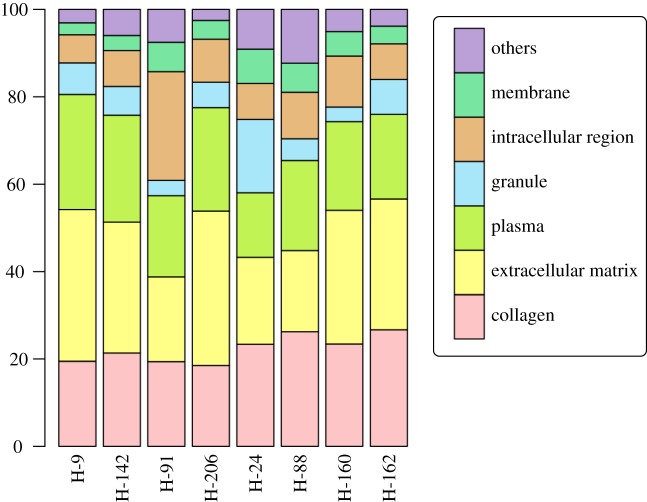
The composition of detected proteins. *X*-axis corresponds to sample ID of each individual.

We applied shotgun proteomics using LC-MS/MS to the skeletal samples excavated from the Hitotsubashi site in Tokyo (AD 1657–1683, Edo period, Japan). The number of detected proteins varied among individuals from 127 to 188 (table 1). There were no significant correlations between the estimated age at death and the number of detected proteins/unique peptides (Pearson’s *r*=−0.4082, *p*=0.3154 for proteins; *r*=−0.3480, *p*=0.3983 for unique peptides). Venn diagrams indicated that infants and adults shared 33 proteins, while adults and elderly adults shared 46 proteins (figure 1*a*). Infants and elderly adults shared 21 proteins. The common proteins between females and males were less than those among males (figure 1*b*). Elderly females shared 25 proteins with adult males and 23 proteins with elderly males. Adult males and elderly males shared 48 proteins. The composition of proteins was shown in figure 2. This indicates that approximately half of total proteins were collagens and extracellular matrix. Plasma proteins accounted for 10–25% of total proteins. Proteins expressed in granules, the intracellular region and membrane were also detected from each bone sample.

**Table 1.** Summary of the proteins extracted from samples. Proteins: number of identified unique proteins from each sample. Peptides: the number of identified unique peptides from each sample. Deamidation (%): the deamidation rate of glutamine calculated using COL1A1 and COL1A2.

sample ID	sex	age (years)	proteins	peptides	deamidation (%)
H-9	—	0.75	173	794	10.1
H-142	—	4	162	497	13.3
H-91	male	29±6.7	158	341	4.1
H-206	male	38±13.1	188	782	9.2
H-24	female	60±13.0	127	387	10.0
H-88	male	60±13.0	175	483	10.0
H-160	male	60±13.0	160	520	10.9
H-162	female	72±12.7	145	554	10.9

With regard to the presence of unique peptides, COL1A1 had the largest number of unique peptides in all individuals, followed by COL1A2. Other collagens, such as COL2A1, COL5A1, COL5A2, COL12A1 and COL22A1, were also detected in almost all samples. Plasma proteins, such as ALB, prothrombin (F2) and coagulation factor IX (F9) were also detected. The top 30 proteins, with the exception of the olfactomedin-like protein (OLFML3), were functionally connected to at least one other protein in the STRING network (electronic supplementary material, figure S1). Osteocalcin (BGLAP), one of the major components of bone, was detected in all samples. Two proteins related to osteoclast regulation were detected in some of the samples: macrophage colony-stimulating factor 1 (CSF1), which plays an essential role in the proliferation and differentiation of osteoclasts [40], and tumour necrosis factor receptor superfamily member 11A (TNFRSF11A), which is essential for RANKL-mediated osteoclastogenesis [41].

### 3.2. Leucocyte-derived proteins in archaeological human bones

We identified proteins derived from leucocytes, such as neutrophils and eosinophils, in archaeological human bones. Warinner *et al.* [42] found leucocyte-derived proteins in human dental calculus; however, to date, no research detecting these proteins in archaeological human bones, including immunological analysis, has been performed. To our knowledge, this is the first study detecting leucocyte-derived proteins in archaeological human bones.

Neutrophils are the most abundant white blood cells and are the first-responders among inflammatory cells to migrate towards the site of inflammation during its beginning phase, particularly as a result of bacterial infection [43]. A variety of neutrophil-derived proteins were detected from the Hitotsubashi samples, including neutrophil defensin 1/3 (DEFA1/3), neutrophil elastase (ELANE), myeloperoxidase (MPO), azurocidin (AZU1) and cathepsin G (CTSG). DEFA1/3, ELANE and MPO are characteristic proteins in azurophilic granules, which are a type of granule found in neutrophils [43,44]. The unique peptides in these proteins were significantly correlated to each other (electronic supplementary material, table S1).

Eosinophils have a key role in allergic and inflammatory processes, including asthma and host resistance to parasites, such as helminths, but they also exhibit antimicrobial activities towards bacterial, viral and protozoan pathogens, and mediate hypersensitivity diseases [45]. In this study, eosinophil peroxidase (EPX) was detected in all samples; EPX is uniquely expressed in eosinophils and not in other cells [45]. Another protein expressed in eosinophil granules, proteoglycan 2 (PRG2), was also detected, and the number of unique peptides was significantly correlated with that of EPX (Pearson’s *r*=0.7782, *p*<0.05). These results indicate the presence of eosinophils in archaeological human bones. Eosinophil-derived neurotoxin (EDN/RNASE2) and eosinophil cationic protein (ECP/RNASE3) were not detected, likely because of the small amount of the proteins or the difficulty of detecting proteins with relatively short sequences [46].

### 3.3. Gene Ontology enrichment analysis

**Figure 3.**
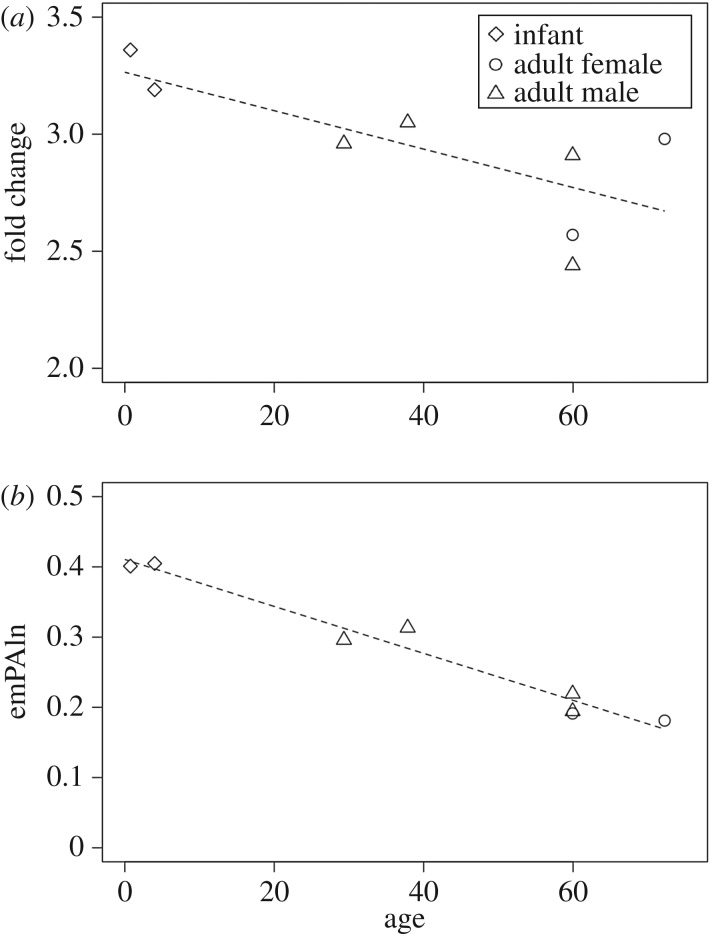
Age-related changes of proteins. (*a*) Relationship between age and fold change of the number of proteins annotated with the GO term ‘developmental process’ (GO: 0032502) (Pearson’s *r*=−0.7386, *p*=0.03638). (*b*) Relationship between age and the normalized emPAI value of AHSG (Pearson’s *r*=−0.9826, *p*=1.295×10^−5^).

We performed GO enrichment analysis in order to estimate what kinds of processes were over-represented. Bone formation processes, such as ‘collagen fibril organization’ and ‘ossification’, were enriched (electronic supplementary material, figure S2). There were also processes associated with non-collagenous proteins (e.g. ‘extracellular structure organization’, ‘extracellular matrix organization’ and ‘cell adhesion’). The processes most enriched in infants were associated with developmental processes, including ‘tissue development’, ‘multicellular organismal development’, ‘single-organism developmental process’, ‘developmental process’ and ‘anatomical structure development’, Age at death was significantly negatively correlated with the fold change of the ‘developmental process’ (Pearson’s *r*=−0.7386, *p*<0.05; figure 3*a*). The numbers of proteins involved in the ‘ossification’ process in infant and adult samples were twice as large as that in the elderly adult (electronic supplementary material, figure S2). In GO Cellular Component terms, the ‘extracellular matrix’ was enriched most remarkably in the infant (electronic supplementary material, figure S3). In GO Molecular Function terms, ‘growth factor binding’ was clearly enriched in the infant (electronic supplementary material, figure S4).

### 3.4. Protein quantitation using exponentially modified protein abundance index

In order to compare the amount of proteins in each sample, we used the emPAI score, one of the most common label-free approaches used for absolute quantification of shotgun proteomics [37]. EmPAI scores of all proteins were normalized by that of COL1A1. The sum of the normalized emPAI scores for non-collagenous proteins showed significantly negative correlation with age (Pearson’s *r*=−0.7088, *p*<0.05). There were proteins with normalized emPAI scores showing a significantly negative correlation with age (table 2). In particular, the normalized emPAI score of alpha-2-HS-glycoprotein (AHSG) showed a strong, significantly negative correlation with age (Pearson’s *r*=−0.9826, *p*=1.295×10^−5^; figure 3*b*). The normalized emPAI scores of ALB, insulin-like growth factor-binding protein 5 (IGFBP5), kininogen-1 (KNG1) and pigment epithelium-derived factor (SERPINF1) also showed a significantly negative correlation with age (electronic supplementary material, figure S5). Among these proteins, AHSG was the only protein whose emPAI score was still statistically significant after multiple test correction (table 2). There were no emPAI scores that significantly and positively correlated with age. AHSG, also known as fetuin-A, is produced not only in the liver but also in osteocytes [47], and is characterized by particularly high mineral affinity. IGFBP5 is an IGF-binding protein involved in the regulation of cell growth and is the most abundant IGFBP stored in bone, where it controls osteoblast differentiation and osteoblast–osteoclast crosstalk [48]. Kininogens are inhibitors of thiol proteases with primarily two isoforms, high-molecular-weight kininogen (HMWK) and low-molecular-weight kininogen (LMWK). HMWK is essential for blood coagulation and the assembly of the kallikrein–kinin system, while LMWK is not involved in blood coagulation. SERPINF1 is a multifunctional secreted protein with anti-angiogenic, anti-tumorigenic and neurotrophic functions. A form of osteogenesis imperfecta, a heritable bone dysplasia characterized by bone fragility and deformity and growth deficiency with a mineralization defect, is caused by mutations in SERPINF1 [49].

**Table 2.** Correlation between normalized emPAI scores and age. *p*-Value after Bonferroni’s correction is marked with an asterisk.

gene name	*p*-value	*p*-value*	Pearson’s *r*
SERPINF1	0.01933536	0.8894264	−0.7912284
ALB	0.0168598	0.775551	−0.8010759
AHSG	1.29×10^−5^	5.95×10^−4^	−0.9826241
KNG1	0.007853061	0.3612408	−0.8477033
IGFBP5	0.001842309	0.08474621	−0.9075273

There are proteins highly expressed only in infants. Periostin (POSTN), formerly named osteoblast-specific factor-2, was originally cloned from a mouse osteoblast cell line [50]. Its function is as a cell-adhesion molecule for preosteoblasts and it is thought to be involved in osteoblast recruitment [50,51]. POSTN was remarkably high in the youngest infant (unique *peptides*=11, normalized *emPAI*=0.0685), although no peptide was found in adult and elderly adult samples. Apolipoprotein A-1 (APOA1) is the major protein component of high density lipoprotein (HDL) in plasma. The expression level of APOA1 in blood has been reported to decrease with age [52,53]. In this study, APOA1 was detected only in the youngest infant (0.75 years old), which is consistent with the previous research [52,53].

## 4. Discussion

### 4.1. The authenticity of the proteomic data

In general, the problems of ancient biomolecule analysis are contamination from the modern environment and the preservation of endogenous molecules. Discerning contaminant molecules from endogenous molecules is especially problematic, particularly for ancient DNA analysis. However, in the case of ancient protein analysis, there is no amplification process (e.g. PCR for DNA); thus, contaminant proteins have less of an effect on the endogenous proteins. In addition, the expression patterns of proteins are tissue-specific (e.g. keratin from human epidermis), making the identification of contaminant proteins easier. The proteomic analysis software Maxquant also provides a list of common protein contaminants [33]; thus, we removed proteins included in the list, with the exception of albumin, which is a common protein detected from archaeological bones [8,35]. To address the issue of endogenous molecule quality, proteomic data from the samples were assessed using two indices: the rate of deamidation and the proportion of type I collagen. Deamidation is an indicator of the preservational quality, and the rate of deamidation of glutamine range is 0–20% in bones of 0–2000 years ago [54]. In this study, the rate of deamidation was 10% on average (range 4–13%; table 1), which is compatible with bones dating *ca* 500 years before present. Type I collagen accounts for 90% of the organic matter of bone, and the collagen triple helix is composed of two alpha 1 chains and one alpha 2 chain. Therefore, it is expected that the proportion of type I collagen is dominant in archaeological bones. In our samples, collagen type I alpha 1 (COL1A1) had the largest number of unique peptides followed by collagen type I alpha 2 (COL1A2). The number of unique COL1A1 peptides showed significant correlation with that of COL1A2 (Pearson’s *r*=0.9715, *p*=5.636×10^−5^). These findings indicate that the proteomics data accurately reflect the composition of the modern bone proteome. In addition, the atomic C/N ratios and gelatin yields, the proxies for the preservation of collagen used in isotope analysis, indicated that the samples from the Hitotsubashi site were well preserved [55]. From these results, we confirmed the preservation of the proteins in our samples.

### 4.2. The presence of leucocyte-derived proteins

An interesting finding in this study was the presence of proteins derived from leucocytes, such as neutrophils and eosinophils. These proteins have not been found in previous studies of archaeological bone [3,8,9,16]. Leucocytes play a critical role in the immune system, so this finding shows the potential of ancient protein analysis for reconstructing the disease, health status and stress profiles of past humans.

These proteins were possibly derived from red bone marrow in the rib samples. Red marrow is haematopoietically active and contains erythrocytes and leucocytes, whereas yellow marrow is made almost entirely of fat, with few haematopoietic elements [56]. All bone marrow is haematopoietic red marrow during infancy. With age, red marrow is gradually converted to the fatty yellow type. By early adulthood, the yellow type makes up two-thirds of the total bone marrow, while red bone marrow remains specifically in the ribs, vertebrae and os coxae [57,58]. In this study we used rib samples, which may explain why we could detect ample leucocyte-derived proteins. For instance, these proteins are scarcely detected in the femur [59].

**Figure 4.**
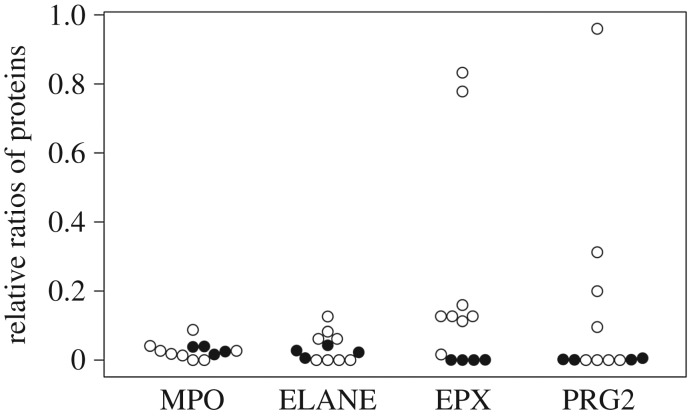
Relative ratios of proteins expressed in neutrophils (MPO and ELANE) and eosinophils (EPX and PRG2). Expression levels of DEFA1/3 were used as a standard. Open circles are from the Hitotsubashi samples. Closed circles are from reference data of The Human Protein Atlas [61].

Although eosinophils constitute only approximately 1–4% of leucocytes of human bone marrow [60], we detected adequate numbers of unique peptides of EPX in all samples. In addition, some individuals showed higher ratios of eosinophil-derived proteins (EPX and PRG2) compared with bone marrow reference data (figure 4). We also found that the number of unique peptides was significantly correlated with that of EPX (Pearson’s *r*=0.7782, *p*<0.05). This may be due to the increase of eosinophils in rib samples. The number of eosinophils increases in association with pathogenesis during asthma, allergic and, particularly, parasitic disease. In the city of Edo, high population pressure led to overcrowding and unhygienic living conditions. It has been reported that there were repeated epidemics among the citizens of Edo at that time [62]. Regarding the Hitotsubashi site, Hirata [20] found that severe cribra orbitalia resulting from iron deficiency anemia was prevalent in the population. In addition, Nagaoka & Hirata [21] reported a relatively short lifespan tendency for the people of Hitotsubashi. Yamamoto [19] reported that a high incidence of dental enamel hypoplasia was found in Hitotsubashi samples. These authors concluded that people residing at the Hitotsubashi site lived under a highly stressful environment [19–21]. According to the historical literature, helminth infection was prevalent during the Edo period [63–65]. These reports support our observation that eosinophil-derived proteins were highly expressed in individuals excavated from the Hitotsubashi site and this may reflect the presence of infectious disease, such as parasite infections.

### 4.3. Age-related changes in the proteins of human bones

The protein profile, structure and composition of bone change with age [66]. Indeed, levels of certain proteins in bone are related to age. For example, the content of non-collagenous proteins, such as AHSG and ALB, decreases with age in bone [67,68]. A recent study sought to determine protein groups whose levels change with development in zebrafish bone [69]. However, whether archaeological bones also show these age-related changes is unknown.

Our analysis indicates, for the first time, that proteomics data from archaeological bones reflect the life stages of individuals. This is supported by three levels of analysis: whole profiling by Venn diagram, the functional protein groups by GO enrichment analysis, and individual protein levels by quantification analysis.

First, the Venn diagram analysis among life stages (infant, adult and elderly adult) showed that the number of shared proteins between infant and elderly adult was the smallest among the three comparisons (figure 1*a*). The number of proteins shared by infant and adult was more than that shared by infant and elderly adult, and proteins were shared most between adult and elderly adult. This indicates that the component of proteomics differs most between infants and elderly adults (figure 1*a*).

Second, GO analysis suggests that the proteomic profile reflects the life stage of individuals. GO Biological Process terms associated with development were most abundant in infant bones (electronic supplementary material, figure S2). Specifically, the age of the individuals was significantly negatively correlated with the fold change of the ‘developmental process’ (figure 3*a*). For the ‘ossification’ GO term, the number of proteins involved in that process in infants and adults was twice as large as that of the elderly adult. In GO Cellular Component terms, the ‘extracellular matrix’ was enriched most remarkably in the infant (electronic supplementary material, figure S3). In GO Molecular Function terms, ‘growth factor binding’ was clearly enriched in the infant (electronic supplementary material, figure S4). These results suggest that infants have more proteins involved in development and growth.

Third, quantitative analysis using emPAI suggests that there are some proteins whose expression undergoes age-related changes. Sum emPAI scores for non-collagenous proteins in total showed significantly negative correlation with age (Pearson’s *r*=−0.7088, *p*<0.05). In regard to each protein, the emPAI score of AHSG correlated most strongly with age (figure 3*b*). The normalized emPAI scores for ALB, IGFBP5, KNG1 and SERPINF1 also negatively correlated with age (electronic supplementary material, figure S5).

It is noteworthy that AHSG, ALB and IGFBP5 reportedly show age-related changes in modern bone. Wilson *et al.* [70] reported that the levels of AHSG and ALB in fetal bone were at least one order of magnitude greater than those of adult cortical bone. Quelch *et al.* [67] reported that AHSG, ALB and sialic acid were present in higher concentrations in neonatal bone compared with bones from children and adults. The concentration of AHSG in neonatal bone was approximately three times higher than that in bone from children, and seven times higher than that in adult bone. The ALB concentration in neonatal bone was one-and-a-half times higher than that in child bone and twice as higher than that in adult bone. With regard to IGFBP5, Mohan *et al.* [71] reported that a decrease in IGFBP5 has been linked to age-related bone loss. Our results from archaeological human bones indicated that the levels of AHSG were correlated most strongly with age; ALB and IGFBP5 also showed a tendency to decrease with age. These results are consistent with the previous research in modern human bone [67,70,71].

Owing to high concentrations of AHSG in fetal serum, multiple functions related to development have been suggested [72,73]. Following collagen, AHSG is one of the most abundant proteins in bone and its ability to bind calcium and other minerals supports the notion that it is essential for osteogenesis [74,75]. Current studies suggest that circulating AHSG is highest during infancy, declines in childhood and varies in adults owing to genetic factors, obesity and diet [73]. AHSG might also be impacted by the natural ageing process [76]. It has been demonstrated that the level of AHSG in bone is also highest in infants and declines with age [67,68,70]. From these findings, we believe our results, indicating high levels of AHSG in infants that decline with age, are consistent and generalizable to other archaeological bones.

### 4.4. Insights of proteomics analysis in archaeological human bones and future perspective

Shotgun proteomics enabled us to analyse more than 100 proteins with accuracy and robustness. Our findings confirmed by multiple proteins that leucocytes such as neutrophils and eosinophils remained in archaeological rib bones. We also found certain proteins to be significantly correlated with age. In addition, we used samples of rib bone for which morphological information related to disease and age did not previously exist. Collectively, our study has demonstrated significant achievement in ancient proteomics, revealing biological processes not visible on the exterior.

Leucocyte-derived proteins provide new insights of paleopathological interest. IgG has been studied by enzyme-linked immunosorbent assay (ELISA) [77], Western blot [78] and LC-MS/MS [18] for detecting the immune responses of the past; however, some studies found it difficult to detect IgG in archaeological bone [6,18]. By contrast, leucocyte-derived proteins such as DEFA1/3 and EPX were prevalently detected in archaeological rib bones in this study. This suggests that these proteins may be better target molecules for studying the immune system.

Furthermore, our results suggest that rib bone is suitable for detecting proteins abundant in bone marrow, particularly for leucocyte proteins. Vertebrae and os coxae are also promising bone elements owing to the remaining red bone marrow [57]. Currently, no method exists to detect an infection in an individual from their bones, except severe stages of some diseases morphologically apparent, such as syphilis and tuberculosis. The particularly prevalent helminths are difficult to detect, because they do not seriously damage the human body. In this study, eosinophils, involved in allergic reactions or parasitic infections, were prevalently detected, which could be related to infectious disease during the Edo period. However, while highly suggestive, our results are insufficient to verify the existence of an infection, thus more rigorous quantitative methods, such as selected reaction monitoring (SRM), would be favourable as a next step.

Our results indicate that AHSG emPAI scores correlate strongly with age. Previous studies have suggested that AHSG exists prevalently in archaeological bone [35], which might suggest that we can estimate life stages of other species or chronologically older samples. There were other proteins that correlated with age or that were expressed only in infants, such as ALB, IGFBP5, POSTN and APOA1. Even though the sample size was small, the data were consistent with previous findings of modern samples. Based on the relative amount of these proteins, we can confirm and further narrow down the estimated age range for each individual, especially when dealing with remains from sub-adults. The full potential of the innovative approach we present here should, however, be fully validated with future research.

The analysis of ancient proteins could provide a useful indicator of stress, disease, starvation, obesity and other kinds of physiological and pathological information. For example, ancient proteins have revealed past diseases such as osteogenic sarcoma and prostate carcinoma [17,79]. Scott *et al.* [80] reported that osteocalcin analysis is promising for detecting stress. The analysis of multiple proteins is preferable to confirm these results.

The field of ancient DNA has entered a new era of genomics and has provided valuable genetic information about past human history [81]; nevertheless, ancient DNA is unable to answer many questions, which makes it difficult to obtain a comprehensive picture of ancient human life. The method of ancient proteomics analysis undoubtedly complements the techniques of ancient DNA analysis. This novel study sheds light on the potential of ancient proteomics for obtaining biological information such as age-related changes or immune system processes, which cannot be extracted from conventional bioarchaeological methods.

## Supplementary Material

Supplementary Materials for Proteomic proling of archeological human bone

## Supplementary Material

Supplementary dataset

## Data accessibility

The mass spectrometry proteomics data have been deposited to the ProteomeXchange Consortium via the PRIDE partner repository [82] with the dataset identifier PXD006256.
