# Supplementary material for: Proteomic profiling of archaeological human bone
Source: R Soc Open Sci. 2017 Jun 7;4(6):161004. doi: 10.1098/rsos.161004 (PMC5493901; doi:10.1098/rsos.161004)
Supplement: Supplementary Materials for Proteomic proling of archeological human bone [file rsos161004supp1.pdf]

Royal Society Open Science

Supplementary Materials for Proteomic profiling of archeological human bone

Rikai Sawafuji[a,b], Enrico Cappellini[c], Tomohito Nagaoka[d], Anna K Fotakis[c], Rosa Rakownikow Jersie-Christensen[e], Jesper V. Olsen[e], Kazuaki Hirata[d], Shintaroh Ueda[a,f,1]

[a] Department of Biological Sciences, Graduate School of Science, the University of Tokyo, 7–3–1 Hongo, Bunkyo-ku, Tokyo 113–0033, Japan.

[b] Present address: Department of Human Biology and Anatomy, Graduate School of Medicine, University of the Ryukyus, 207 Uehara, Nishihara, Nakagami, Okinawa, 903–0215, Japan.

[c] Centre for GeoGenetics, Natural History Museum of Denmark, University of Copenhagen, ster Voldgade 5–7, 1350, Copenhagen, Denmark.

[d] Department of Anatomy, St. Marianna University School of Medicine, 2–16–1 Sugao, Miyamae-ku, Kawasaki, Kanagawa 216–8511, Japan.

[e] Novo Nordisk Foundation Center for Protein Research, Faculty of Health Sciences, University of Copenhagen, Blegdamsvej 3b, 2200 Copenhagen, Denmark.

[f] School of Medicine, Hangzhou Normal University, No.58, Haishu Rd, Cangqian, Yuhang District, Hangzhou, Zhejiang 311121, China.

[1] Corresponding author: To whom correspondence should be addressed. E-mail:sueda@bs.s.u-tokyo.ac.jp

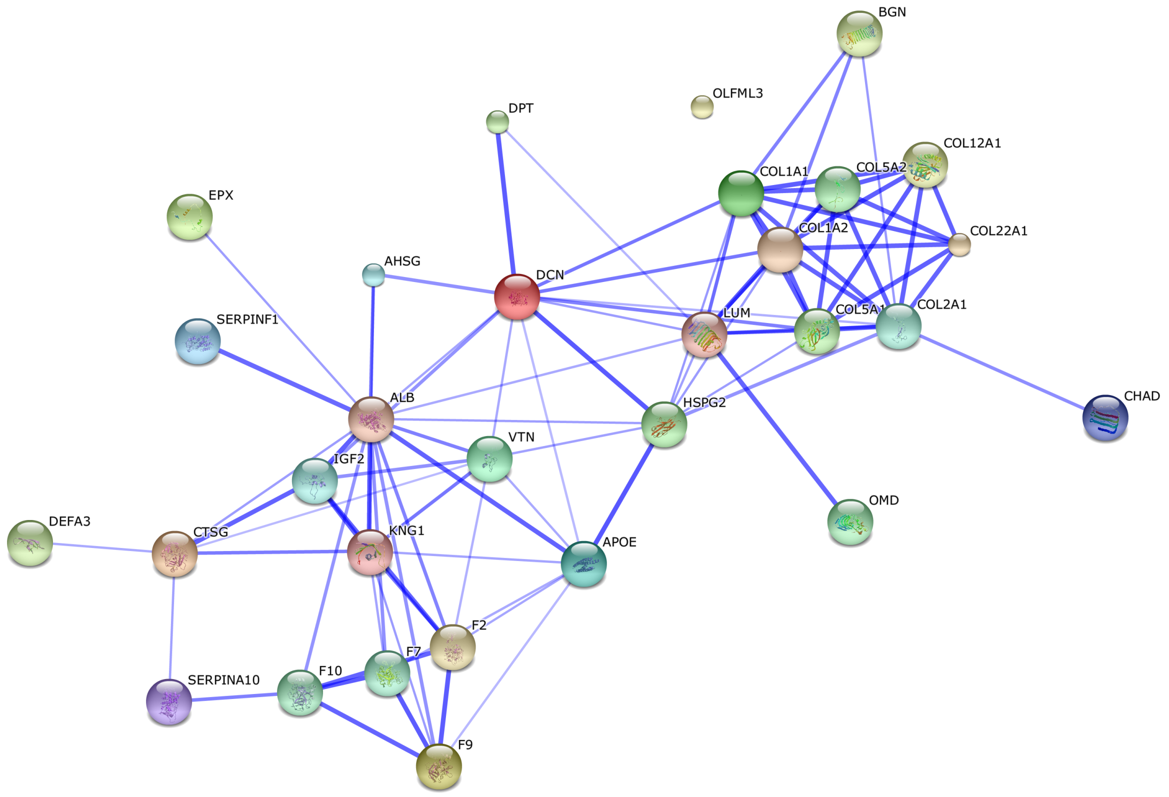

Figure S1: STRING network of top 30 proteins. Blue lines between nodes represent functional associations between proteins. The thickness of the lines indicates the strength of confidence in the association reported.

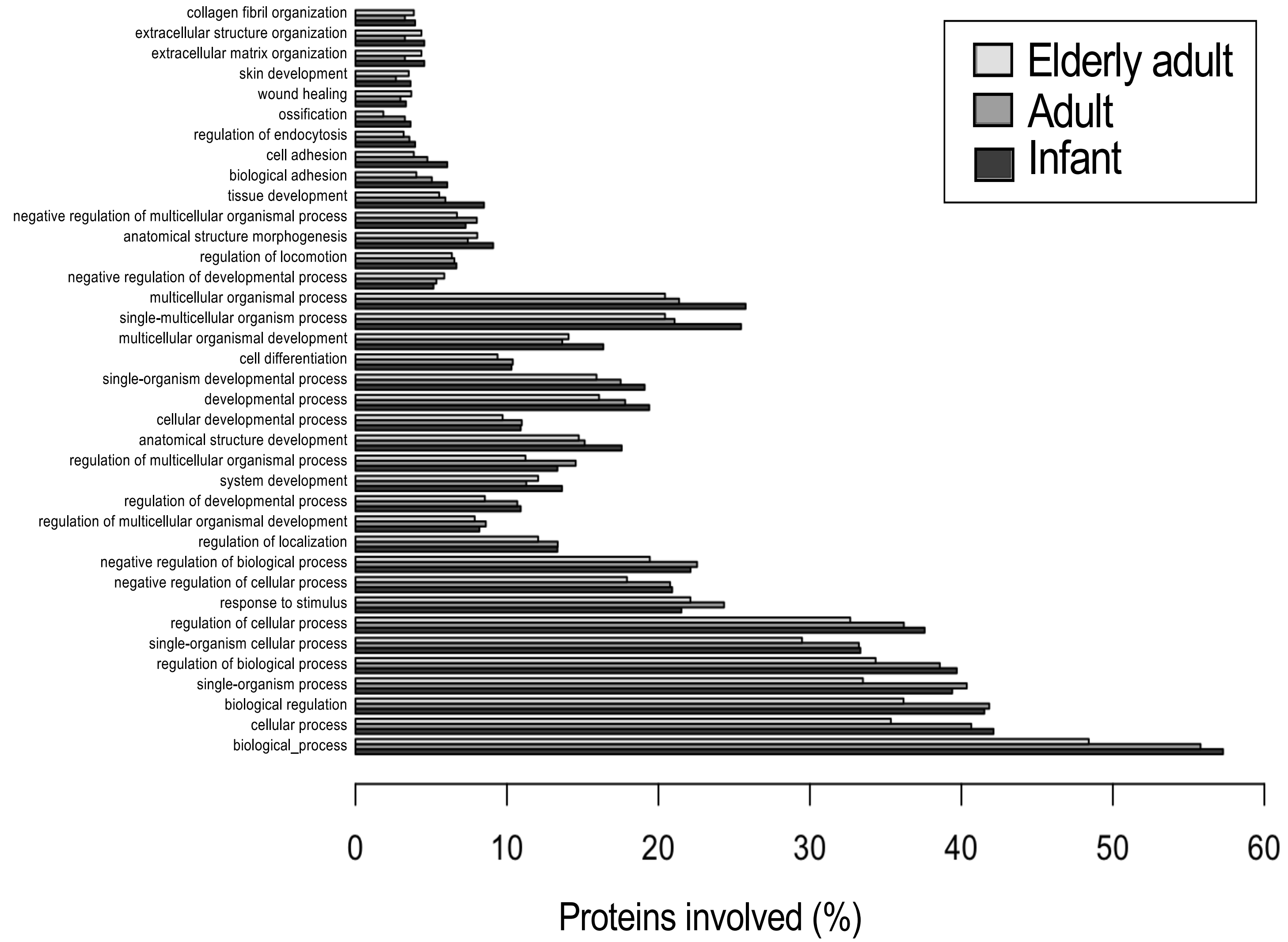

Figure S2: Enrichment GO term of the Biological Process. GO terms for which at least two of all samples were significant ($p < 0.05$) in enrichment analysis are shown.

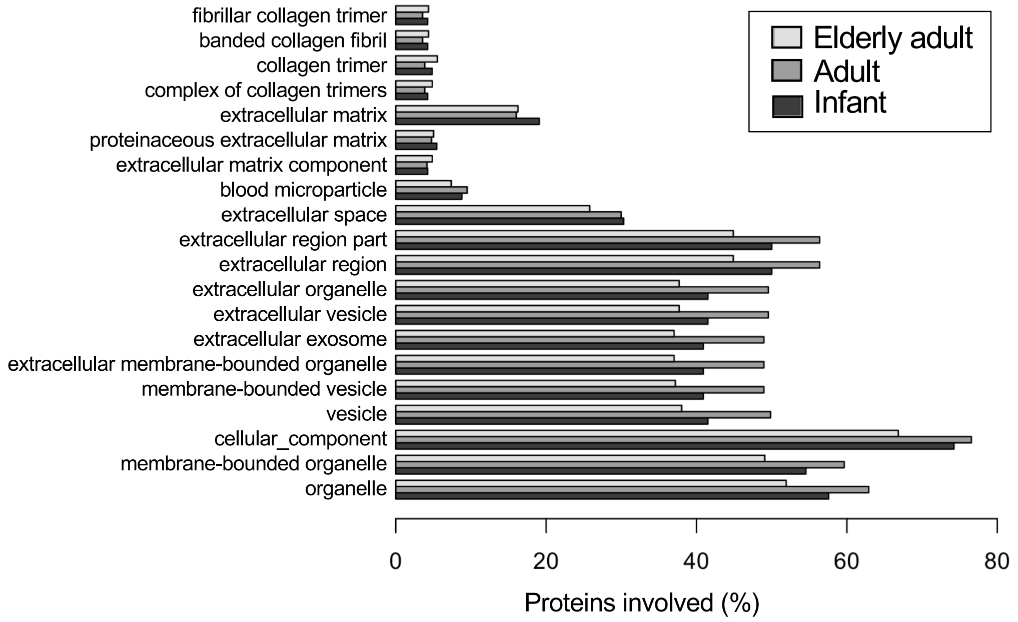

Figure S3: Enrichment GO term of the Cellular Component. GO terms for which at least two of all samples were significant ($p < 0.05$) in the enrichment analysis are shown.

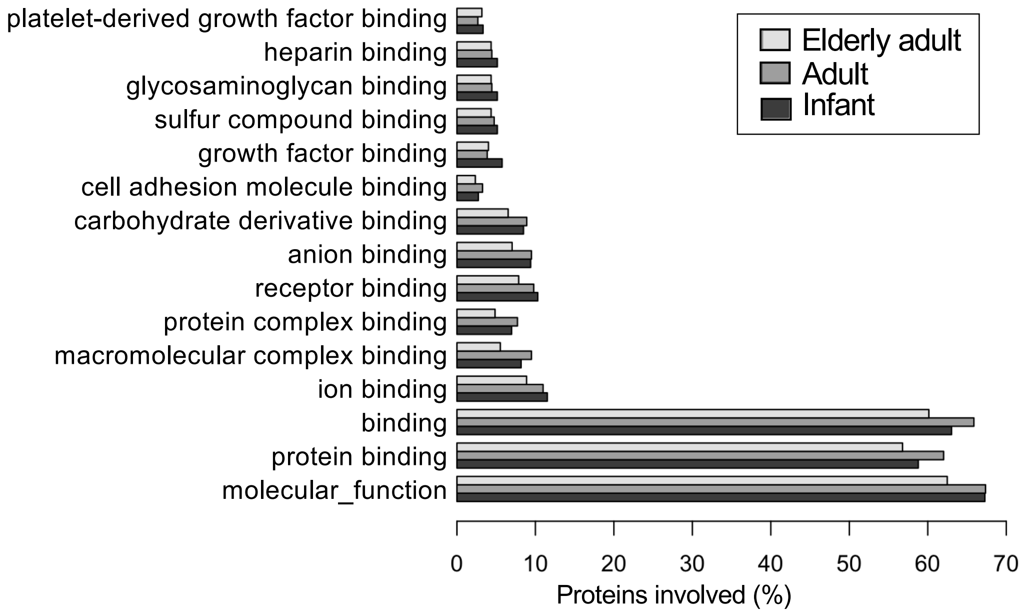

Figure S4: Enrichment GO term of the Molecular Function. GO terms for which at least two of all samples were significant ($p < 0.05$) in the enrichment analysis are shown.

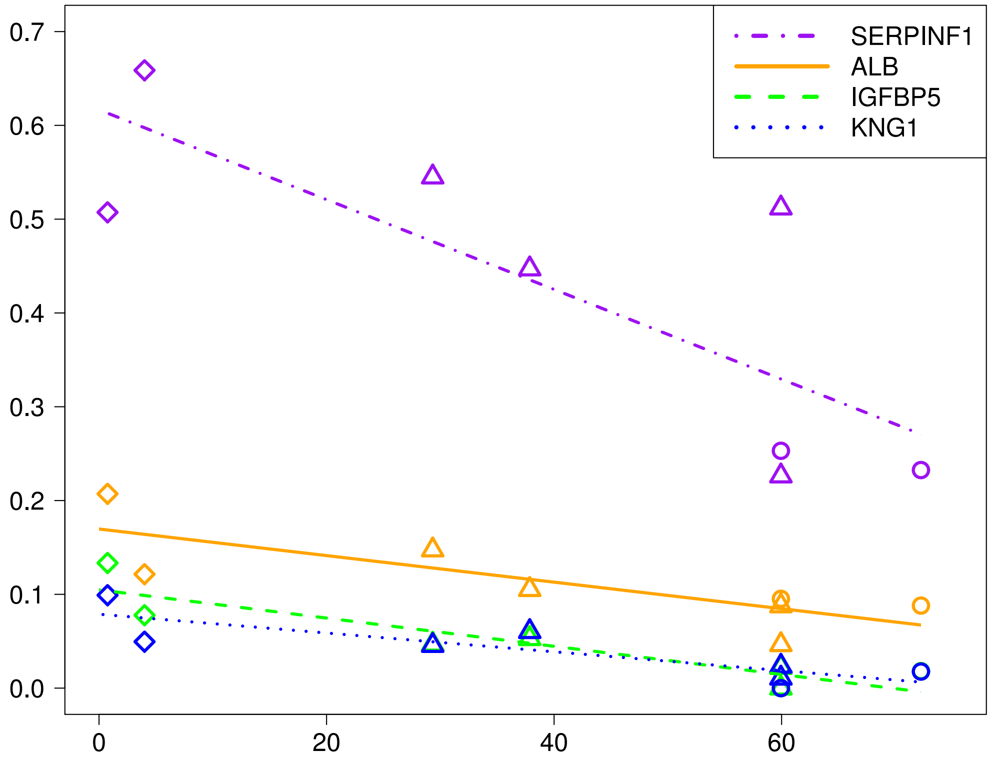

Figure S5: Relationship between age and the normalized emPAI value of proteins correlated with age (See Table 2). Diamonds, infants; circles, adult females; triangles, adult males.

Table S1: Correlation between the unique peptides of neutrophil-derived proteins. *P* values for pearson's correlation coefficient were shown.

Gene name	MPO	ELANE
DEFA3	0.03905	0.003096
MPO	—	0.02797
ELANE	0.02797	—
